# CADDSuite – a workflow-enabled suite of open-source tools for drug discovery

**DOI:** 10.1186/1758-2946-4-S1-O2

**Published:** 2012-05-01

**Authors:** Oliver Kohlbacher

**Affiliations:** 1 Applied Bioinformatics, Center for Bioinformatics, University of Tübingen, Germany

## 

We present an open-source suite of tools for computer-aided drug design, CADDSuite, built upon our molecular modeling framework BALL. CADDSuite provides a wide range of tools for structure preparation, docking, QSAR and related topics.

IMGDock is a novel docking tool combining heuristic search strategies and a grid-based scoring function. We demonstrate that it yields results comparable other current docking tools on popular docking benchmark sets. It can be easily combined with TagRes, a tool for target-specific rescoring that allows a more accurate estimation of binding free energies within a specific target family.

All tools of the CADDSuite, including IMGDock and TagRes, are open-source software (under the GNU Public License). Tools of the CADDSuite share a common binary interface, a common data exchange format and thus easily integrate into distributed computing environments. We show how they can be used from KNIME and how KNIME workflows can then be executed on distributed resources, in particular Galaxy and WS-PGRADE for grid computing.

